# Barriers and Facilitators Associated With Remote Concussion Physical Assessments From the Perspectives of Clinicians and People Living With Workplace Concussions: Focus Group Study

**DOI:** 10.2196/56158

**Published:** 2024-11-13

**Authors:** Keely Barnes, Heidi Sveistrup, Motahareh Karimijashni, Mark Bayley, Mary Egan, Martin Bilodeau, Michel Rathbone, Monica Taljaard, Shawn Marshall

**Affiliations:** 1 School of Rehabilitation Sciences Faculty of Health Sciences University of Ottawa Ottawa, ON Canada; 2 Bruyère Research Institute Ottawa, ON Canada; 3 Clinical Epidemiology Program Ottawa Hospital Research Institute Ottawa, ON Canada; 4 School of Human Kinetics Faculty of Health Sciences University of Ottawa Ottawa, ON Canada; 5 Department of Systems and Computer Engineering Carleton University Ottawa, ON Canada; 6 KITE Research Institute Toronto Rehabilitation Institute University Health Network Toronto, ON Canada; 7 Division of Physical Medicine and Rehabilitation Temerty Faculty of Medicine University of Toronto Toronto, ON Canada; 8 Department of Medicine, Division of Neurology Faculty of Health Sciences McMaster University Hamilton, ON Canada; 9 School of Epidemiology and Public Health University of Ottawa Ottawa, ON Canada; 10 Department of Medicine University of Ottawa Ottawa, ON Canada

**Keywords:** remote care, mild traumatic brain injury, telehealth, assessment, workplace injury, concussion, telemedicine, brain injury

## Abstract

**Background:**

Evaluating the clinical status of concussions using virtual platforms has become increasingly common. While virtual approaches to care are useful, there is limited information regarding the barriers and facilitators associated with a virtual concussion assessment.

**Objective:**

This study aims to identify the barriers and facilitators associated with engaging in virtual concussion assessments from the perspective of people living with workplace concussions; identify the barriers and facilitators to completing virtual concussion assessments from the perspectives of clinicians; and identify the clinical measures related to 4 clinical domains that would be most appropriate in virtual practice: general neurological examination and vestibular, oculomotor, and cervical spine assessment. We also evaluated effort.

**Methods:**

Separate online focus groups were conducted with expert concussion clinicians and people living with workplace concussions. A moderator led the focus groups using a semistructured interview guide that targeted a discussion of participants’ experiences with virtual assessments. The discussions were recorded, transcribed, and analyzed by 2 reviewers using content analysis. Barriers and facilitators associated with completing the physical concussion examination were categorized based on the domain of the concussion examination and more general barriers and facilitators. Clinician-selected measures believed to work best in a virtual practice were described using frequency counts.

**Results:**

A total of 4 focus groups with 15 people living with workplace concussions and 3 focus groups with 14 clinicians were completed using Microsoft Teams. Barriers were identified, such as triggering of symptoms associated with completing an assessment over video (mentioned 13/162 (8%) and 9/201 (4%) of the time for patient and clinician participants, respectively); challenges with location and setup (mentioned 16/162 (10%) of the time for patient participants); communication (mentioned 34/162 (21%) and 9/201 (4%) of the time for patient and clinician participants, respectively); and safety concerns (mentioned 11/162 (7%) of the time for patient and 15/201 (7%) for clinician participants). Facilitators were identified, such as having access to support (mentioned 42/154 (27%) and 21/151 (14%) of the time for patient and clinician participants, respectively); implementing symptom management strategies throughout the assessment (mentioned 11/154 (7%) of the time for patient participants); and having access to resources (mentioned 25/151 (17%) of the time for clinician participants). From the perspective of the clinician participants included in this study, the clinical measures recommended most for a virtual practice were finger to nose testing; balance testing; the Vestibular/Ocular Motor Screening tool; saccades; and cervical spine range of motion within their respective domains (ie, neurological examination, vestibular, oculomotor, and cervical spine assessment).

**Conclusions:**

Virtual assessments appear to be useful for both people living with workplace concussions and clinicians. While barriers were identified, such as challenges associated with exposure to screens, virtual assessments have benefits such as improved access to care. The clinician-selected measures that were considered best in a virtual practice will be investigated in an upcoming evaluative study.

**International Registered Report Identifier (IRRID):**

RR2-10.2196/40446

## Introduction

### Background

Concussions result from indirect or direct impacts to the head and usually lead to neurological symptoms [1]. While most individuals who sustain a concussion recover within days of the injury, an estimated 10% to 20% of individuals experience persistent symptoms after the injury requiring intervention [2]. Following a concussion sustained in the workplace, there is an increased likelihood of reporting persistent symptoms [3,4]. This may be attributed to several factors, including access to worker’s compensation, fear of returning to the site of the injury [3], and the demands of work that can exacerbate symptoms. Timely access to care can be challenging for workers in remote and rural communities due to poor access to specialized clinicians [5-8]. The COVID-19 pandemic further presented challenges to accessing in-person care for workplace injuries [9,10].

Virtual care, the remote delivery of health care services that may address some of these challenges, often uses visual and audio communication or videoconferencing technology [11,12]. For patients with a concussion, virtual care could improve access to care [7,13-16] and has been shown to be beneficial from the perspective of clinicians, although not without limitations [17]. A recent study by van Ierssel et al [17] explored the perspectives of 25 clinicians on assessing and managing concussions through virtual care approaches. They reported that clinicians believed that virtual care could be beneficial for monitoring the functional status at follow-up, for screening patients, and for identifying those who may require further in-person assessment. Moreover, participants did not believe care to be jeopardized by virtual follow-up assessments. In general, the literature supports the view that clinicians recognize advantages to using virtual care approaches for concussion assessment and management, but there is a need to further explore the effectiveness of virtual assessment in the context of concussion care.

There is limited information on the barriers and facilitators associated with virtual care from the perspectives of people with a concussion. One study [18] explored virtual care experiences from the perspectives of patients, caregivers, and health care providers in the geriatric medicine setting. Uncertainty about accuracy (ie, the ability to validly identify deficits) was reported as a concern with patients and caregivers, who questioned whether a comprehensive understanding of patient status was captured [18]. However, patient and caregiver participants in this study highlighted the benefits, such as improved access to care and engagement with caregivers in appointments, emphasizing that a hybrid approach to care may be superior to virtual-only approaches [18]. However, with regard to concussion care, experiences may differ from those in the geriatric medicine setting. People with concussion may experience greater fatigue when compared to other populations due to screen exposure [19]. Furthermore, most concussions occur in younger people (<30 years) when compared to the geriatric population [20], who are potentially more comfortable and less intimidated by the technology involved [21]. Both the clinician and patient perspectives are important for a comprehensive understanding of the potential usefulness of the technology [11], and therefore, a detailed exploration of experiences associated with virtual assessments from the perspectives of people living with workplace concussion is needed.

There is a critical need to identify the barriers and facilitators associated with the main domains of the physical examination (ie, neurological examination, vestibular, oculomotor, and cervical spine), when completing a hands-on comprehensive assessment is not possible [22-24]. There is an additional need to evaluate the effort used during an assessment, which is particularly the case in the context of injured workers, as the presence of and access to compensation following a work-related injury could influence performance (if low effort is used) and subsequent findings on an examination [25]. While the evaluation of effort is not a clinical domain, it is considered a component of the examination used to interpret the clinical results and is therefore presented as a separate “domain” throughout this paper.

### Objectives

This study aimed to (1) explore the barriers and facilitators related to engaging in virtual concussion assessments from the perspective of people living with workplace concussions; (2) document the barriers and facilitators associated with completing a virtual concussion examination (ie, neurological examination, vestibular, oculomotor, and cervical spine domains and effort) as identified by concussion expert clinicians with experience in completing virtual assessments; and (3) document concussion expert clinicians’ opinions regarding which clinical measures from the aforementioned domains work best in a virtual practice. This study focused on the assessment of physical domains due to substantial adaptations required to administer these measures virtually and due to individual interpretations of how best to administer these when compared with measurements of emotional or cognitive health.

## Methods

### Overview

In this paper, we report on a part of a mixed methods study. The full protocol [26] and initial findings from surveys and a working group to identify targeted clinical measures [27] have been recently published. This paper reports on focus group discussions related to the previously identified clinical measures. This paper follows the guidelines outlined by the Standards for Reporting Qualitative Research (Multimedia Appendix 1).

### Ethical Considerations

Research ethics approval was obtained from the Ottawa Health Sciences Network Research Ethics Board (20210575-01H), the University of Ottawa Board of Ethics (H-02-22-7611), and the Bruyère Research Ethics Board (M16-22-006). Verbal consent over the telephone was obtained from all participants before participation in the focus groups. Privacy and confidentiality were maintained by using deidentified data. Participants were provided with a CAD $30 (US $22) gift card for their participation in the focus group.

### Participants and Recruitment

Patient participants were recruited through the Ontario Workers Network clinic at the Ottawa Hospital. Eligible patient participants were adults (aged >18 years) who had attended at least one initial or follow-up virtual assessment following a workplace concussion injury through a videoconferencing platform. Purposive sampling was used to identify these eligible patient participants [28]. The sampling strategy was designed to ensure adequate distribution of specific characteristics such as patient age and sex. Eligible participants were approached by telephone and were provided with a description of the aims of the study before consenting. We attempted to recruit at least 5 participants per group [29,30]; however, some participants did not attend the scheduled group or had to cancel, so the groups ranged from 2 to 5 participants. We attempted to include a diverse group of participants in terms of age, date since injury, and sex.

Eligible clinician participants were Canadian-practicing clinicians (ie, neurologists, physiatrists, family medicine physicians, sports medicine physicians, physiotherapists, athletic therapists, and kinesiologists) with expertise in treating concussions (not specifically workplace concussion) and with experience in completing virtual concussion assessments. Eligible clinician participants were recruited through targeted emails and outreach to rehabilitation clinics across Canada. Purposive sampling was used [26] to ensure the distribution of certain characteristics, such as clinician profession. Similar to the patient participant groups, we attempted to recruit 5 to 7 clinicians per group; however, due to scheduling challenges, the groups ranged from 4 to 5 participants. We attempted to include a diverse group of clinicians in terms of clinical profession.

### Data Collection

In a previous paper [24], we surveyed clinicians concerning their use of clinical measures in their in-person practice. The clinicians rank-ordered the measures based on relevant measures to their in-person practice within the following domains: neurological examination, vestibular, oculomotor, cervical spine, and effort assessment. We then documented the perceived feasibility of administering these measures virtually, based on expert clinician opinion [27]. The measures identified in this work were further discussed in this focus group study.

Focus groups were completed over a videoconferencing platform (Microsoft Teams) from August 2022 to January 2023. Consenting participants attended one focus group (either a clinician group or a patient group). Semistructured guides (refer to the study by Barnes et al [26]) were used to direct the discussion for both clinician and patient focus groups. The guides were reviewed and revised by the study team before the commencement of the focus groups to confirm the appropriate wording of the questions and ensure the questions captured the aspects that the team was aiming to explore. The patient interview guide consisted of 9 questions regarding practical and technical barriers and facilitators to undergoing a virtual assessment and sought feedback and recommendations for virtual assessments. The clinician interview guide included 12 questions related to (1) practical and technical barriers and facilitators to virtual assessments and (2) domain-specific questions related to the neurological examination, vestibular, oculomotor, and cervical spine domains and effort assessment, as well as those prompting the participants to discuss and select the outcome measures [24,25] within each clinical domain that they felt would work best in a virtual practice.

Focus groups were moderated by KB, a female registered kinesiologist. KB held a master’s degree in human kinetics and was a PhD candidate in rehabilitation sciences at the time of the study. KB worked as a kinesiologist for 2 years both in a complex care clinic where Workplace Safety and Insurance Board concussion-injured workers were assessed and treated as well as in home-based kinesiology services. A notetaker (MK) was present to prevent loss of data, record nonverbal expressions and key discussion points to add further context to the recording, and aid with technical issues. The focus groups were audio-video recorded and transcribed verbatim by KB.

All participants completed a demographic form following the completion of the focus group. Questions related to age, sex, work status, virtual assessments completed after the concussion, and symptoms were completed for patient participants. Questions related to age, sex, clinical profession, clinical experience, volume of practice of patients with a concussion, virtual assessments completed in the last year, and self-perceived competency associated with completing the in-person and virtual assessment were completed for clinician participants.

### Data Analysis

#### Clinician and Patient Participant Identification of Barriers and Facilitators

**Figure 1:**
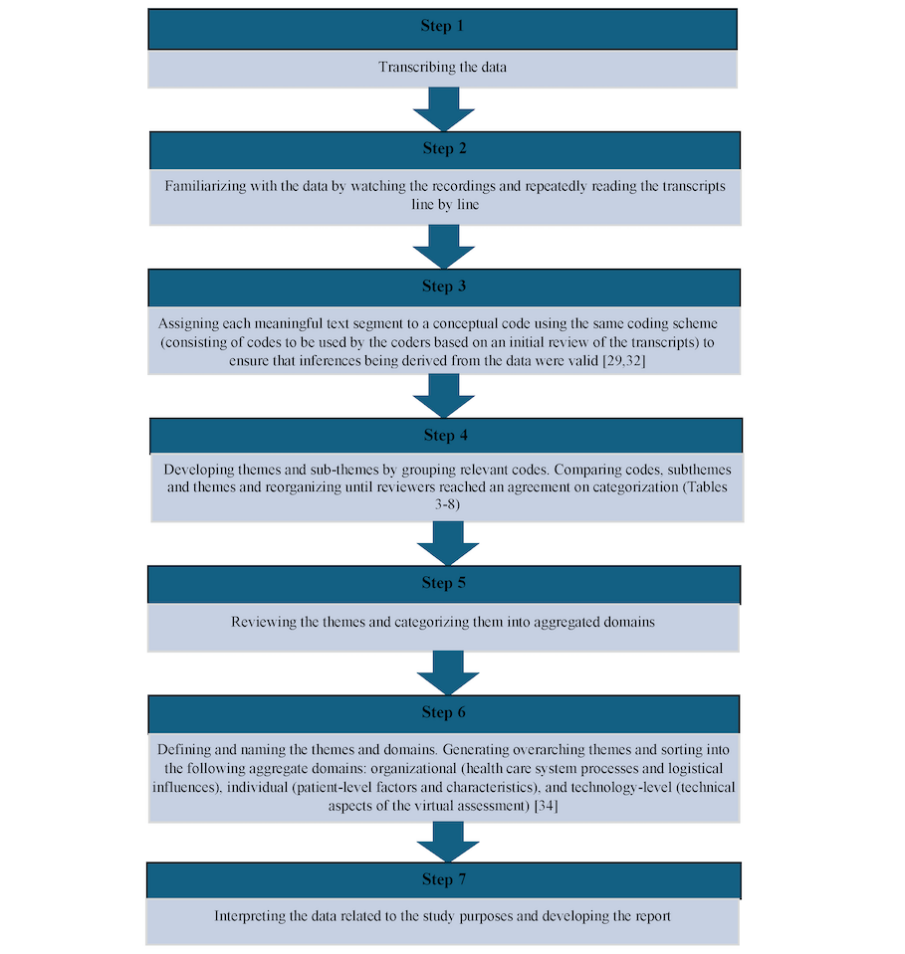
Content analysis process flow diagram reflecting the 7 steps that led to the development of results.

Two reviewers (KB and MK) used inductive qualitative content analysis [31] in NVivo qualitative data analysis software (version 12; QSR International Inc). Analysis started simultaneously with data collection so that data saturation (the point at which no new themes were identified) could be monitored. The analytic process is presented in Figure 1 [29,32-34].

To ensure that inferences being derived from the data were valid, the coders analyzed the data using the same coding scheme (consisting of codes to be used by the reviewers based on an initial review of the transcripts) [29,32]. They met weekly to peer review data analyses and discuss interpretations until reaching consensus. Frequency counts were generated for each theme by tabulating the number of times each theme was identified in the transcripts, and the percentage of times each theme was reported by the participants was presented.

The clinician subthemes were further categorized into the predefined categories (domain-specific and general categories). Domain specific was defined as any barrier or facilitator that was discussed in relation to specific measures under the following domains: neurological examination, vestibular, oculomotor, cervical spine, and effort. General was defined as any barrier or facilitator that relates to the virtual assessment not specific to the domains. The patient participant subthemes were all categorized as general barriers and facilitators. NVivo was used to calculate Cohen κ coefficients to determine the agreement between the 2 coders for all the codes.

#### Clinician Participant Selection of Clinical Measures

Prereading materials were provided, to the clinician participants only, before the focus group. These materials contained descriptions of the clinical measures identified by clinicians in an earlier study [27] to be discussed in the focus groups, YouTube links with video demonstrations of the measures, and a summary of select psychometric properties of each measure. Details of these materials are described elsewhere [27]. From this list, the clinicians selected the ones that they perceived would provide the most information when administered virtually. The 2 reviewers independently documented the frequency of the clinician selection of each clinical measure.

## Results

### Focus Group Structure

Overall, 7 focus groups (4 with people living with workplace concussions and 3 with clinicians) were completed. Saturation was reached after 3 groups for both the patient and clinician groups; however, as we experienced challenges recruiting male participants, we conducted one additional male-only focus group. This allowed us to have 2 mixed-sex focus groups, a female-only focus group, and a male-only focus group. Focus groups were made up of 2 to 5 participants. A total of 15 people with workplace concussions and 14 clinicians participated in the focus groups. The focus group discussion lasted between 30 and 90 minutes each as some groups contained only 2 participants and some groups contained up to 5. Cohen κ was calculated as 0.80 across all groups (considered substantial agreement) for all codes for both the clinician and patient participant focus groups.

### Participants

#### Patient Participants

Table 1 presents the demographic information of the patient participants. The age range of the patient participants was between 20 and 59 (mean 40.9, SD 13.1) years. Most participants were female (11/15, 73%). All participants were experiencing persistent symptoms after workplace concussion injury at the time of their virtual assessment. Injury dates upon participation in the focus group ranged from 9 months to approximately 4 years after a concussion. All participants reported physical symptoms as most common (15/15, 100%), followed by cognitive (6/15, 40%) and emotional symptoms (2/15, 13%).

**Table 1:** Patient participant demographic characteristics (n=15).

Characteristics		Participants, n (%)
**Age range (y)**		
	20-29	3 (20)
	30-39	4 (27)
	40-49	5 (33)
	50-59	3 (20)
	>60	0 (0)
**Sex**		
	Female	11 (73)
	Male	4 (27)
**Current work status**		
	Off work	2 (13)
	Full return to work	5 (33)
	Modified return to work, same occupation	6 (40)
	Modified return to work, different occupation	2 (13)
**Virtual assessments completed after the concussion**		
	1-5	7 (47)
	6-10	4 (27)
	>10	4 (27)
**Currently experienced symptoms**		
	Somatic symptoms (headaches, tinnitus, vision blurriness, fatigue, nausea, migraines, and dizziness)	15 (100)
	Emotional symptoms (overstimulation, anxiety, anger, and frustration)	2 (13)
	Cognitive symptoms (slow thinking and expression of thoughts, memory, issues processing information, and inability to concentrate)	6 (40)

#### Clinician Participants

Table 2 presents the demographic information for the clinician participants. Each focus group consisted of a mix of clinicians. A total of 8 physicians (ie, sports medicine physicians, physiatrists, and a family medicine physician) and 6 other clinicians (ie, athletic therapists and physiotherapists) participated. The clinicians’ virtual assessment experience ranged from ≤10 virtual assessments completed in the last year to over 20 completed virtual assessments completed in the last year. Most clinicians self-reported their competency of completing the in-person and virtual assessment as “strongly competent” and “competent,” respectively.

**Table 2:** Clinician participant demographic characteristics (n=14).

Characteristics			Participants, n (%)
**Age range (y)**			
	20-29	1 (7)	
	30-39	5 (36)	
	40-49	2 (14)	
	50-59	3 (21)	
	>60	3 (21)	
**Sex**			
	Female	6 (43)	
	Male	8 (57)	
**Clinical profession**			
	Physiotherapist	4 (29)	
	Physiatrist	5 (36)	
	Athletic therapist	2 (14)	
	Sports medicine physician	2 (14)	
	Family medicine physician	1 (7)	
**Clinical experience (y)**			
	1-5	3 (21)	
	5-10	4 (29)	
	>10	7 (50)	
**Current volume of practice of patients with a concussion**			
	0-25	5 (36)	
	25-50	2 (14)	
	>50	6 (43)	
	Did not respond	1 (7)	
**Virtual assessments completed in the last year**			
	0-10	3 (21)	
	10-20	1 (7)	
	>20	7 (50)	
	Unsure	3 (21)	
**Competency of in-person neurological assessments**			
	Strongly incompetent	0 (0)	
	Incompetent	0 (0)	
	Neutral	1 (7)	
	Competent	4 (29)	
	Strongly competent	9 (64)	
**Competency of virtual neurological assessments**			
	Strongly incompetent	0 (0)	
	Incompetent	1 (7)	
	Neutral	3 (21)	
	Competent	8 (57)	
	Strongly competent	2 (14)	

### Themes

#### Barriers

##### Overview

Tables 3 and 4 present the barriers for both the patient and clinician participants. Quotes are represented as numbers in the text within each aggregate category. For example, “P12-Q1” refers to quote number 1 from Patient participant 12 or “C2-Q7” refers to quote number 7 from Clinician participant 2. For the clinicians only, categorization of the subthemes (domain specific or general) are presented. Percentage of times each theme was reported by the participants is presented. This was calculated by dividing the frequency of times a specific theme was mentioned by participants by the total number of times all themes were mentioned by participants. For example, location and setup at home was identified as a barrier by patient participants (ie, due to limited space, noise at home, being in a different province, etc) 9.9% (16/162) of the time. Clinical process–related factors were identified as a barrier by clinician participants 10.5% (21/201) of the time. Rankings associated with the barriers reported by the patient and clinician participants are presented in Multimedia Appendix 2.

**Table 3:** Patient and clinician participant barriers associated with virtual concussion assessments.

Aggregated domains, overarching themes, and subthemes				Patients					Clinicians				
				Identified by the patient participant			Patients reported (out of a total of 162 themes mentioned), n (%)		Identified by the clinician participant		Clinicians reported (out of a total of 201 themes mentioned), n (%)		Clinician categories

**Organizational level**													
	**Environmental** **setup**												
		Location and setup at home			✓	16 (9.9)		—^a^		—		—	
	**Clinical process–related factors**					—				21 (10.5)			
		Lack administration support			—	—		✓				General	
		Time			—	—		✓				Domain specific: cervical; neurological	
**Individual level**													
	**Communication and engagement**					34 (21)				9 (4.5)			
		Challenges with communication			✓			✓				Domain specific: neurological	
		Challenges building rapport and connecting with clinicians			✓			✓				General	
	**Triggering physical and emotional symptoms**					13 (8)				9 (4.5)			
		Screens trigger physical symptoms			✓			✓				General	
		Emotional			✓			—		—		—	
	**Safety**					11 (6.8)				15 (7.5)			
		Unease working to limit ability			✓			—		—		—	
		Feelings of tests being safer in person			✓			—		—		—	
		Safety			—	—		✓				Domain specific: vestibular; neurological	
	**Comfort**									9 (4.5)			
		Patient comfort and preference			—	—		✓				Domain specific: effort	
		Psychological impact			—	—		✓				General	
	**Environmental setup**												
		Environmental and patient setup			—	—		✓		16 (8)		Domain specific: vestibular; oculomotor; neurological; effort	
**Technology level**													
	**Technology and internet**					32 (19.7)				41 (20.4)			
		Health care system barriers			✓			—		—		—	
		Issues or lack comfort with technology and the internet			✓			—		—		—	
		Format of required documents			✓			—		—		—	
		Technical and internet issues			—	—		✓				Domain specific: oculomotor; neurological	
		Camera and device limitations			—	—		✓				Domain specific: vestibular; oculomotor; neurological	
	**Accuracy and completeness of physical examination**					56 (34.6)				53 (26.4)			
		Incomplete evaluation or lack accuracy			✓			—		—		—	
		Lacking physical contact			✓			—		—		—	
		Unable to complete assessments in entirety			—	—		✓				Domain specific: vestibular; oculomotor; cervical; neurological	
		Lack accuracy			—	—		✓				Domain specific: vestibular; oculomotor; cervical; neurological	
		Unable to assess certain conditions and subtleties			—	—		✓				Domain specific: vestibular; oculomotor; cervical; neurological	
	**Visibility**												
		Observability	—			—		✓		28 (13.9)		Domain specific: vestibular; oculomotor; cervical; neurological	

^a^Not identified by participants.

**Table 4:** Patient and clinician participant barriers quotes associated with virtual concussion assessments.

Aggregated domains, overarching themes, and subthemes				Quotes
**Organizational level**				
	**Environmental** **setup**			
		Location and setup at home	“A lot of the times they couldn’t do my assessment virtually because I lived in a different province” [P12^a^-Q1^b^]	
	Clinical process–related factors			“But think about the time that it takes to explain to somebody, try to see what it is that you’re doing. You know it’s, it takes far more time than seeing them in person.” [C2^c^-Q7]b
**Individual level**				
	Communication and engagement			“Harder to explain um what was going on. I also have speech issues so trying to express sometimes over virtual is difficult.” [P6-Q4] “I found the virtual assessment created a bit of a barrier on creating relationships, though, and you weren’t able to as easily connect or especially if it is your first time seeing them, athlete or patient.” [C3-Q11]
	Triggering physical and emotional symptoms			“My big, bothersome side effects was ah like technology. Just the audio like the kind of the sound that comes out of the audio really irritated me. And with the visual like the screen that would really drive my eyes and my head crazy, so it wasn’t really convenient for, to be in front of a screen trying to recover from a concussion.” [P14-Q3] “Depending on how symptomatic they are, screen time isn’t great for them. So, um you know, decreased tolerance to screen time is one of the things and you might not be able to make it through a full assessment.” [C6-Q10]
	Safety			“Once COVID hit, we were all like on video with the clinic and it was a little bit harder because I’ve fallen a lot. Umm. I’m in a walker now. A walker. Um. And sometimes there aren’t people here at the house, so if I do fall I, it’s a little scarier in a way.” [P1-Q2] “If they had really poor balance, you’re not there to guard them as a physio. We’re always kind of told to guard when you’re doing that kind of stuff and they have nobody else there to help guard then that’s a problem.” [C9-Q8]
	Comfort			“When you think about effort, it’s like, well, part of it is that the individual has to be comfortable, right, in the virtual assessment... I wouldn’t draw any conclusions around effort from a virtual assessment.” [C4-Q12]
	Environmental setup			“A barrier for gait and tandem gait is kind of having enough space where you can see them walking on the screen too. So probably back to that camera umm set up and everything and then making sure there’s enough space in their house so they can actually do a long enough walk that you can see any abnormalities too.” [C6-Q9]
**Technology level**				
	Technology and internet			“The Internet stuff because I have a hard time doing computers right now and due to vision loss and stuff and I just forget how to do it. So, um that’s something that’s, if I didn’t have somebody here, I wouldn’t have been, I wouldn’t have been able to do it.” [P3-Q6] “I find the eye tests are the most challenging and mainly it’s you know, it’s whose finger to follow is 1 but also the resolution especially if depending on the quality of their camera it’s the hardest and the closer they get to the screen of course the more blurry it becomes to them also, so it’s really hard.” [C10-Q15]
	Accuracy and completeness of physical examination			“I have only had one in person um assessment with both OT and the doctor. And it was like very hands on compared to any of the other assessments before. The other assessments like that were online kind of just felt like we were going through a checklist to make sure like you know, nothing’s like terrible condition, but the one in person, for sure, that felt longer and more in depth.” [P8-Q5] “If you’re gonna do this, you’re gonna have them walk by the camera and you sort of say, ok, now aim the camera down at your feet as you’re walking or that, go back far enough there just really isn’t enough space for that, camera is not wide enough and whatnot or goes low enough to really get a good assessment. You can get a generally gestalt of it, but I don’t think it’s an accurate assessment.” [C1-Q13]
	Visibility			“It’s the visibility of, you know, trying to count errors on camera.” [C2-Q14]

^a^P: patient participant.

^b^Q: quote.

^c^C: clinician participant.

##### Patient Participant Barriers

###### Organizational Level

Patient participants reported some issues with being in a province different from that of the assessing clinician. For example, some professional associations require clinicians to be licensed to practice in the province in which the patient is located for the virtual assessment (the patient may be located in Quebec and the clinician in Ontario). Participants further voiced concerns regarding privacy associated with using a videoconferencing platform such as the fear of people overhearing conversations and concerns regarding the security of the videoconferencing platform (Q1).

###### Individual Level

Patient participants expressed safety concerns (Q2). Participants believed that certain components of the assessment would feel safer when done in person, specifically the balance tests. They believed this would have an impact on the results; participants felt that they could “perform better” and “push out of their comfort zone” with the in-person assessments when compared to the virtual assessments.

Patient participants felt that virtual assessments could activate physical and emotional symptoms. The use of screens was commonly reported to trigger physical concussion symptoms (headaches, dizziness, vision issues, etc; Q3). Feelings of isolation and depression could be increased during virtual assessment: “you do feel very isolated” and “you are all alone and it gets very depressing.”

Patient participants also reported challenges communicating issues related to their condition and challenges building rapport with their clinicians through videoconferencing platforms (Q4).

###### Technology Level

A barrier identified included the perspective that less accurate information is obtained with the virtual assessment and the perspective that the virtual assessment is incomplete. The participants perceived that clinicians were unable to see subtle issues associated with their condition, such as “eye twitches” or “tremors.” Participants perceived the in-person examination to feel more in-depth and hands-on, whereas the virtual assessment felt unfinished (Q5). Furthermore, participants expressed a lack of physical contact with the virtual assessment, which seemed to be particularly noticeable for the cervical spine examination.

Participants reported issues with the internet connection and with the videoconferencing platforms used for the virtual assessment. Different clinicians using different platforms, videoconferencing platforms that are difficult to use, and the format of the required documents, such as needing to complete web-based questionnaires, were identified as challenges to completing the virtual assessment (Q6).

##### Clinician Participant Barriers

###### Organization Level

Lack of time (due to the additional time required to set the patient up correctly on the screen in a virtual appointment) and lengthy clinical measures were identified as domain-specific barriers (Q7). Challenges due to the lack of administrative support to aid with the virtual assessment process was identified as a barrier, which also relates to the processes in the clinical setting.

###### Individual Level

Safety concerns were expressed with all balance measures and components of the neurological examination (eg, pronator drift due to a need for the patient to close their eyes during the test and functional squat) by clinician participants (Q8).

Environmental and patient setup was identified as a domain-specific barrier. Specifically, clinicians reported challenges with the patients’ environment, such as lacking space, and challenges getting patients in appropriate positions to complete clinical measures (Q9).

Clinicians reported that computer screens can trigger physical concussion symptoms, which makes it challenging to complete a comprehensive assessment virtually, representing a general barrier. Clinicians expressed that patients have minimal tolerance to screens, which leads to a need to take frequent breaks (Q10).

Clinicians identified challenges with communicating with patients through screens, including challenges with explaining how to complete a measure virtually and challenges associated with building rapport and forming connections with patients, reflecting a general barrier (Q11).

A lack of patient comfort with the virtual assessment and clinician stress or frustration associated with completing the virtual assessment due to technical challenges were identified as barriers. The importance of patient comfort was particularly relevant to the discussion regarding the evaluation of effort (observing if the patient is performing at optimal capacity) in a virtual assessment. Clinicians reported that due to the lack of comfort and confounding factors (ie, fatigue, symptom aggravation associated with the screen, and unclear picture), it would be very challenging to draw conclusions regarding the effort used in a virtual assessment (Q12).

###### Technology Level

A domain-specific barrier identified by clinician participants included an inability to complete the physical examination in its entirety. For example, clinicians reported an inability to complete a full cranial nerve examination (a part of the neurological examination) virtually and an inability to touch a patient’s neck when completing cervical spine palpation. Clinicians also questioned accuracy for certain measures (such as the modified Balance Error Scoring System and oculomotor tests) when administered virtually (Q13). Furthermore, clinicians expressed challenges associated with identifying subtle deficits experienced by patients and an inability to assess comorbid conditions. Most of these were limitations identified due to the quality of current technology (clarity of the image on the screen).

Clinicians identified an inability to get an understanding of the full picture, such as missing body language cues due to only being able to see a patient’s upper body. Camera limitations were discussed (Q14).

Camera, device, and internet issues were identified as general barriers to completion of the virtual assessment. Unstable internet, the use of different devices (particularly mobile phones), and positioning of the camera were all barriers identified that could influence the quality of the virtual assessment (Q15).

#### Facilitators

Tables 5 and 6 present the facilitators for both the patient and clinician participants. Rankings associated with the facilitators reported by the patient and clinician participants are presented in Multimedia Appendix 3. A figure presentation of the overarching themes, subthemes, and codes is presented in Multimedia Appendix 4 (patient participants) and Multimedia Appendix 5 (clinician participants). Definitions of the subthemes associated with Tables 3 and 4 are presented in Multimedia Appendices 6 and 7, respectively.

Tables 7 and 8 present the clinician- and patient participant–identified benefits associated with the virtual concussion assessment. While not necessarily facilitators to the use of virtual assessments, the participants identified some benefits to this approach. The information is presented in the same format as has been for the barriers and facilitators, as described earlier.

**Table 5:** Patient- and clinician-participant facilitators associated with virtual concussion assessment.

Aggregated domains, overarching themes, subthemes			Patients			Clinicians		
			Identified by patient participants	Patients reported (out of a total of 154 themes mentioned), n (%)	Identified by clinician participants		Clinicians reported (out of a total of 151 themes mentioned), n (%)	Clinician categories

**Organizational level**								
	**Format of delivered care and material**			23 (14.9)			29 (19.2)	
		Use of functions available on the virtual platform	✓		—^a^		—	—
		Introduction to technology and resources	✓		✓			Domain specific: vestibular; oculomotor; cervical; neurological
		Integration and patient selection of virtual	✓		✓			General
	**Support**			42 (27.3)			21 (13.9)	
		Opportunity to involve more care team members	✓		—		—	—
		Easy contact with clinician or technical support	✓		—		—	—
		Support	—	—	✓			Domain specific: vestibular; oculomotor; neurological
	**Use of resources**							
		Use of resources to complete assessment	—	—	✓		25 (16.6)	Domain specific: vestibular; oculomotor; cervical; neurological
**Individual level**								
	**Symptom management**							
		Offering symptom management strategies	✓	11 (7.1)	—		—	—
	**Support**			42 (27.3)			21 (13.9)	
		Home support	✓		—		—	—
		Support	—	—	✓			Domain specific: vestibular; oculomotor; neurological
	**Environmental setup**							
		Environmental and patient setup	—	—	✓		16 (10.6)	Domain specific: vestibular; oculomotor; neurological; effort
**Technology level**								
	**Use of measure to identify gross deficits**							
		Use of measure as global screen and rely on subjective information	—	—	✓		24 (15.9)	Domain specific: vestibular; oculomotor; cervical; neurological

^a^Not identified by participants.

**Table 6:** Patient- and clinician-participant facilitators quotes associated with virtual concussion assessment.

Aggregated domains, overarching themes, subthemes					Quotes
**Organizational level**					
	Format of delivered care and material			One thing I would suggest is, for things like the neurology appointment, I know I think it would be good if someone could just go um be comfortable just for your appointment, be comfortable. We might need to do this, this, because then, for me, it sounds like for you know, we wanna appreciate the person’s time and also like I’m setting up and making sure things are like, you know, whatever, like, please if I need to do this so having knowing kind of what your spatial requirements are gonna be might be a good idea. Just so that it can help us.” [P2^a^-Q17^b^] “I find that a lot of times the patient gets on to the appointments virtually and they have this sense of anxiety because they don’t know what they’re going to go through in the assessment. They don’t know how long it’s going to last. They don’t know how taxing is going to be for those who are severely concussed it can be incredibly taxing. So, I find just having a basic outline that we’ll be doing some history taking some brief history taking some measurements range of motion and that sort of thing, Balance testing, whatever it is we’re going to be testing just to give them an advance.” [C5^c^-Q22]	
	Support			“But I do find that when you are having technical difficulties, that calling them and they’ll send you a new link or whatever, or they’ll even call you on the phone umm to do your appointments just by voice, which helps me a lot as well.” [P3-Q16] “I’ve done this where an occupational therapist helped me with the eye exam. It was so much. I felt so much more confident. In what we were testing.” [C11-Q24]	
	Use of resources			“The only way you could do this would be with some sort of app, that sort of, uh, can detect that that, that, that pupil or whatnot and then track it for you on the screen as you’re, they’re doing it.” [C1-Q23]	
**Individual level**					
	Symptom management			“During our appointments, we were taught about the blue light ah and certain glasses we can wear during virtual, and then we made-up a rule cause I was always very stubborn at first with my injury and I really like to make eye contact through virtual. I thought it made it look more professional and how it should be. And they told me you don’t need to do that because you are a patient. And so, we did 20 seconds looking at the, looking at the person speaking to you and then nobody will be offended if you look 2 minutes at your wall. So, 20 seconds, 2 minutes. Whenever you start feeling symptoms.” [P7-Q19]	
	Support			“I still have to do the balancing stuff, but I always make sure that somebody is here with me um that my partner is here with me so he can, if something happens.” [P3-Q20] “For some patients, if they have like a, you know, a partner or family member or somebody that you know, you can just ask to come for the physical exam, right? You ask them to just, you know, bring them along. And that helps with some of the safety aspects of it.” [C4-Q26]	
	Environmental setup			“I think sometimes actually just having like a shoe box and a laptop is pretty helpful cause the shoebox lets the laptop be up at more eye height.” [C5-Q27]	
**Technology level**					
	**Use of measure to identify gross deficits**				
		Use of measure as global screen and rely on subjective information	I would say I would say that like eye movements was is really like in my practice a screen to make sure that they have eye movement, didn’t have some kind of odd, you know like palsy, right. But beyond that, it just really you can’t really interpret much more. Virtual.” [C4-Q29]		

^a^P: patient participant.

^b^Q: quote.

^c^C: clinician participant.

**Table 7:** Patient and clinician participant benefits associated with virtual concussion assessments.

Aggregated domains, overarching themes, and subthemes				Patients					Clinicians				
				Identified by patient participants		Patients reported (out of a total of 80 themes mentioned), n (%)		Identified by clinician participants			Clinicians reported (out of a total of 22 themes mentioned), n (%)		Clinician categories

**Organizational level**													
	**Access**					36 (45)					15 (68)			
		Improved access	—		—		✓					General	
		Cost saving	✓				—			—		—	
		Travel	✓				—			—		—	
**Technology level**													
	**Convenience, comfort, and ease**												
		Sense of it being easier and more convenient at home	✓		44 (55)		✓			7 (31.8)		General	

**Table 8:** Patient and clinician participant benefits quotes associated with virtual concussion assessments.

Aggregated domains, overarching themes, and subthemes			Quotes
**Organizational level**			
	Access		“I also had difficult to, traveling the distance because I live a far ways away. So it was, you know, it was nicer to have the virtual just because I didn’t have to travel.” [P6^a^-Q9^b^] “I found that virtual assessments also allowed for patients who couldn’t necessarily drive due to symptoms it facilitates having quicker access as well as, you know, being at least able to check in on them.” [C3^c^-Q25]
	**Convenience, comfort, and ease**		
		Sense of it being easier and more convenient at home	“it’s convenient. It was very convenient to take it in the house and not have to go anywhere and they like again, it was a negative partly, but also a positive was the comfort I felt in my own home. I did feel better, my symptoms were I’d say better than they would have been in the hospital. And. Yeah. So, I think that it was like I, yeah, yeah, I felt I feel safer and more comfortable in my home.” [P11-Q21] “It could be easier to reach out to them a little bit earlier in the process because there aren’t those barriers are of, you know, having to book an in-person appointment, them having to find transportation or having to find support to be brought in. So, they’re in the comfort of their own homes and it’s better for them in terms of their recovery because for a lot of these patients coming in is like a 1/2 day or a full day process.” [C11-Q28]

^a^P: patient participant.

^b^Q: quote.

^c^C: clinician participant.

##### Patient-Participant Facilitators and Benefits

###### Organizational Level

A facilitator identified by the participants included having another health care professional in person with them (if in clinic), such as an occupational therapist, while the physician completed the virtual examination. Participants expressed that having easy access to technical support, when needed, would be helpful in troubleshooting technical difficulties and easing feelings of anxiety (Q16).

Having a choice of device used in the virtual assessment, being prepared in advance of the assessment, and using the features that videoconferencing offers (such as the record function) were facilitators identified by the patient participants that all relate to the process and format in which the assessment is delivered. Specifically, participants reported that having the ability to choose the device and screen size used for the virtual assessment would encourage use of the virtual assessment. Participants felt that having at least one in-person assessment, reflecting a hybrid approach, was helpful to enhance their feelings of comfort with the virtual assessment. Advanced preparation was another key facilitator identified. Participants felt that being contacted before the assessment and being provided with information regarding what to expect would be helpful (Q17).

Having easy access to care was a benefit of virtual assessment use. Participants valued having access to care particularly when they were unable to attend in-person assessments due to various reasons (COVID-19 pandemic restrictions, comorbidities, unable to drive, etc; Q18).

###### Individual Level

Implementing symptom management strategies throughout the virtual assessment was identified as a facilitator. When clinicians offered screen breaks or recommended symptom management strategies to use during the assessment, such as using blue light glasses, the virtual assessments were more manageable for participants (Q19).

Having equipment such as a chair or another person (a partner or family member) at home were facilitators related to both the individual- and organization-level aggregate domains identified by patient participants. Participants reported feeling safe completing their assessments at home with these safety measures in place (Q20).

###### Technology Level

A sense of ease, comfort, and control with virtual assessments was expressed as a benefit. Specifically, feeling that care was not jeopardized when assessed virtually and a sense of virtual assessments being more convenient, comfortable, and requiring less energy were facilitators identified (Q21). Furthermore, the participants felt that they could be more independent with the virtual approach (eg, not rely on others to drive to be able to attend an assessment) and could control their environments better at home when compared to a hospital or clinic setting.

##### Clinician-Participant Facilitators and Benefits

###### Organization Level

Adequate preparation and integration of the virtual assessment with the in-person assessment (hybrid approach to care where the virtual option is available, if needed) were identified domain-specific facilitators. Clinicians expressed that sending information in advance of the assessment and informing patients of what to expect improved ease of completing a virtual assessment (Q22). In addition, having a backup in case the primary connection for the virtual assessment failed would help with the flow of the virtual assessment. These facilitators relate to the format in which the assessment and material needed for the assessment are delivered.

Using resources to support administration of certain measures virtually, such as mobile apps that can measure cervical spine angles or eye movements or systems that could analyze vestibular deficits, were identified as domain-specific facilitators by clinicians (Q23). For example, there are apps (such as HeadCheck) that clinicians reported using in their virtual practice that provide objective information on eye movements.

A domain-specific facilitator identified by the clinician participants included having access to clinical support in person. Clinicians reported that this would be particularly helpful due to safety concerns and due to challenges seeing subtleties over camera (Q24).

Clinicians reported the benefit of improved access to care that virtual care provides. Clinicians perceived that virtual assessment approaches could eliminate the need for patients to drive to assessment centers (or rely on a family member to drive) and perceived that this could facilitate easier and quicker access to care for the patient (Q25).

###### Individual Level

Similar to patient participants, clinicians expressed that holding onto a physical support (eg, chair, wall, and another person) facilitated the administration of certain measures virtually and was expressed as useful for most domains of the physical examination. Furthermore, access to emotional and technical support (a family member or partner) at home could help with the virtual assessment process. This also allows clinicians to gain the family member perspective, which could aid in getting collateral information and obtaining a more comprehensive understanding of clinical status (Q26).

Having an appropriate space to complete the virtual assessment at home and appropriately setting up the patient on the screen were identified as domain-specific facilitators. Clinician participants identified strategies, such as placing the computer on a shoebox and asking the patient to stand in a corner to complete certain measures virtually such as balance tests, would facilitate completion of the physical examination (Q27).

###### Technology Level

Clinicians reported a sense of ease, comfort, and convenience with virtual assessments. Particularly, they expressed that the virtual assessment allows patients to be in the comfort of their homes and eliminates some of the obstacles that need to be overcome to get to assessment centers (time, driving, and costs), which relates to a general benefit (Q28).

Clinician participants reported that using most of the measures to identify gross deficits (rather than specific deficits) or that using some of the measures for subjective information rather than objective information was a facilitator to its use in a virtual context (Q29). For example, when using the modified Balance Error Scoring System, clinicians reported that counting errors is a challenge over camera, so the measure is administered to identify gross balance deficits rather than identify subtle issues. Similarly, when administering the Vestibular/Ocular Motor Screening (VOMS) tool, clinicians rely on subjective reports of symptom changes and do not use this measure to identify specific ocular or vestibular issues.

### Clinician Selection of Measures That Would Work Best in Virtual Assessments

The frequency of selection of each clinical measure is presented in Table 9. Within their respective domains, clinicians identified the finger to nose test, balance testing, the VOMS tool, saccades, and cervical spine range of motion as the measures that would work best and give the most relevant information in their virtual practice out of the list of measures provided.

**Table 9:** Clinician selection of measures that would work best in a virtual practice within the respective domains.

Domain and measures			Description of measures		Selection, n (%)
**Neurological examination**					
	Coordination: finger to nose; rapid, alternating movements	Finger to nose: patient extends arm and touches examiner’s outstretched finger, and then touches patient’s nose Rapid alternating movements: patient alternately taps the back and palm surface of one hand onto the other hand; toe tapping: patient repeatedly taps foot on floor [35]		9 (64)	
	Motor (pronator drift and functional squat)	Pronator drift: stretch both arms forward, with palms facing upward, and eyes closed [35] Functional squat: observe and perform 10 body-weight squats [36]		3 (21)	
	Cranial nerve	I: Smell object II: Read the Snellen chart (a chart with varying-sized letters; cover one eye). Ask the patient to look at your eyes, place hands on both sides of the patient’s head with the index fingers extended, ask the patient to indicate which finger you are moving (left or right) Fundoscopy examination: clinician views the eye using ophthalmoscope III, IV, and VI: eye movements; pursuit (follow moving target), convergence (eyes move at the same time, such as observing an object nearing the patient’s nose), and saccades (alternate gaze between right and left and up and down targets) V: facial sensation; light touch and pinprick both sides; clench teeth, and open jaw against resistance VII: facial movements (show teeth, whistle, and close eyes tightly) VIII: hearing test (tuning fork), test balance, and gait test for nystagmus (beating or twitching of eyes) IX and X: voice (say “ah” with a tongue depressor), swallowing, cough, and gag reflex XI: shrug shoulders and turning the head left and right (against resistance) XII: open the mouth and stick out the tongue [35]		1 (7)	
**Vestibular**					
	Balance (feet together, single leg stance, and tandem stance)	Feet together: standing with feet together; time-based tests Single leg stance: standing on one leg and eyes open and eyes closed; time-based tests Tandem stance: stand in heel to toe stance and eyes closed; time-based test [37]		9 (64)	
	VOMS^a^	Smooth pursuits: the patient maintains focus on the target as the examiner moves the target smoothly in the horizontal and vertical direction Saccades: the examiner holds 2 single points horizontally. Instruct the patient to move their eyes as quickly as possible from point to point. Repeat the test with 2 points held vertically Convergence: the patient focuses on a target and slowly brings it toward the tip of their nose Vestibular ocular reflex: the patient is asked to rotate their head horizontally while maintaining focus on the target. The test is repeated vertically Visual motion sensitivity: the patient holds an arm outstretched and focuses on their thumbs. The patient rotates, together as a unit, their head, eyes, and trunk to the right and left [38]		9 (64)	
	Romberg	Stand with feet together on a flat, hard surface. The examinee is asked to stand still and keep their eyes open for approximately 30 seconds. Next, the examinee is asked to close their eyes and stand for 30 seconds; time-based test [35]		3 (21)	
	VOR^b^ test	Move the head quickly side to side; repeat up and down [35]		3 (21)	
	Gait or tandem gait	Observe the patient walk. Observe the heel to toe walk [35]		1 (7)	
	mBESS^c^	Single leg stance (stand on one leg), tandem (stand in the heel to toe position), and double leg stance (stand with feet together) on flat ground and foam surfaces, with eyes closed. The examiner records errors during each condition (open eyes, lift hands off hips, etc) [39]		0 (0)	
**Oculomotor**					
	Saccades	Rapid movement of eyes between fixed points. Instruct the patient to look from one target to the other as quickly as possible—vertical and horizontal [40]		7 (50)	
	Smooth pursuits	Pursuits are performed in an H pattern. Participants track the object [41]		5 (36)	
	Convergence	Use a pencil, placed just above the nose between the eyes. Move the target toward the patient at a rate of approximately 1-2 cm/s, encouraging them to keep the target single. Measure the patient’s reported subjective break (target becomes double) in centimeters. Measure in centimeters [42]		2 (14)	
**Cervical**					
	Range of motion	Active range of motion: head turn left, right, up, down, and side flexion left and right [37]		13 (93)	
	Self-palpation	Patient feels with fingers and hands during physical examination of the neck [37]		2 (14)	

^a^VOMS: Vestibular/Ocular Motor Screening.

^b^VOR: vestibular ocular reflex.

^c^mBESS: modified Balance Error Scoring System.

## Discussion

### Principal Findings

This study used focus groups to identify barriers and facilitators associated with virtual concussion assessments. To our knowledge, this work is the first to explore the barriers and facilitators associated with virtual concussion assessments from the perspectives of people living with workplace concussions. The findings of this study indicate that a hybrid approach (first assessment completed in person and follow-ups completed virtually) to concussion assessment is preferred by both people living with workplace concussions and clinicians. It appears that the virtual approach to concussion assessment is of value, highlighting a need to further explore and consolidate information on the effectiveness of the clinical measures used in concussion assessments when administered virtually. People living with workplace concussions seem to prefer having the initial assessment with the care team in person, followed by virtual assessments as needed. Virtual assessments were perceived as most appropriate for follow-ups and for a global screen of deficits rather than for identifying specific deficits.

In this study, overlapping themes were identified for both clinician and patient participants. Interestingly, both clinicians and patients commented on difficulties associated with completing the required documents (questionnaires) virtually. Both expressed a need to verbally complete the required documents with the patients over the phone or have support in place so that they have help to complete them before the virtual assessment. This is in line with Beaton et al [43], who documented that the preferred method of information delivery for people living with a concussion is in auditory format.

Both clinicians and people living with workplace concussions highlighted that inaccurate values may be obtained with the virtual assessment, which was the most common barrier identified (identified by patient and clinician participants approximately 35% and 26% of the time, respectively). Objectivity in assessments conducted using virtual care is a challenge, particularly with the lack of clarity when observing certain movements through videoconferencing, which in turn influences clinicians’ abilities to adequately assess patients [24]. Virtual care has been used for the management of concussions; however, an identified limitation is the challenge associated with the remote physical examination [24]. Subtle abnormalities such as those observed during objective oculomotor assessments could be difficult to capture through videoconferencing [7]. Furthermore, a neurological examination in its entirety is not currently feasible through videoconferencing [44,45]. Particularly, a complete evaluation of the cranial nerves (fundoscopy) cannot be completed along with motor and sensory function, cervical spine palpation, strength testing, muscle reflexes, and neuro-otology maneuvers such as those for vertigo [7,15,36,46]. Both clinicians and patients expressed challenges associated with using technology, including the virtual approach to assessment that can be symptom-triggering, safety concerns with the virtual assessment in relation to potential for falling, challenges with the setup at home, and barriers associated with communicating and establishing a clinician-patient rapport through a screen.

This study identified technical, individual, and organizational-level barriers that are in line with some of the literature on virtual care in people living with neurological conditions. Ownsworth et al [47] reported limitations associated with videoconferencing specifically, which include issues with technology and incompatibility for the patient (inability to use technology). Technical issues such as poor video or audio quality due to either the varying quality of devices used or Wi-Fi signal strength and difficulty placing the camera are identified barriers specific to videoconferencing [11]. Logistical challenges when a caregiver is not present at home to aid with the virtual session contributes to the nonfeasibility of virtual care approaches [13]. Concerns of lack of security and concerns regarding privacy in terms of family members overhearing sessions are described barriers [11], which was a concern expressed by patient participants in this study. Consistent with the findings of this study, the literature documented that the lack of resources including the lack of access to the administrative or skilled personnel support needed and lack of time, as virtual assessments are perceived to take more time to appropriately set up the patient, influence the feasibility and use of videoconferencing approaches [48]. A limitation associated with virtual assessments identified by the participants in this study includes lack of physical presence [47]. Lack of physical presence has been documented to be associated with challenges in terms of building rapport when not face to face as well as an inability to gain a full understanding of the patient’s status [47]. The patient-clinician relationship may be jeopardized if technologies are used due to the difficulties associated with building trust through screens and the challenges of clear communication [15,44,46].

From a facilitation standpoint, both the clinicians and people living with workplace concussions in this study highlighted the convenience and improved access that virtual assessments provide. Both groups emphasized the need to have appropriate supports and preparation in place; however, only the patient participants identified this as the most common facilitator (identified approximately 27% of the time). The patient participants in this study identified the importance of having easy access to technical support. This is in line with Hale-Gallardo et al [13] who reported that virtual care coordinators and technical clinicians are viewed as resources that could facilitate the adoption of technology-based care in clinical practice. Having assistants help set up the system is a necessary facilitator to videoconferencing [16]. Family support is additionally viewed as a facilitator due to the help they provided with the technical and clinical aspects of videoconferencing [11]. The increased likelihood of family members being present during videoconferencing is an advantage, which could aid with gathering information [49]. This is also helpful to aid with remembering recommendations and next steps of care. Clinician participants identified the format of the assessment and materials (adequate preparation and offering virtual assessments as a complement to in-person assessments) as the most common facilitator (identified approximately 19% of the time). Development of clear instructions and advanced planning to prepare for issues are all necessary facilitators to videoconferencing [16]. The importance of having an initial in-person touchpoint and preference for the hybrid approach was highlighted in the literature. This approach enhances the comfort of the patient with the clinician [11]. Clinician and patient understanding of the benefits of virtual approaches to care, which includes cost savings, autonomy for the user, and reduced travel and wait times act as facilitators for the use of technology-based approaches to assessment [11,16,46].

Several unique findings of this study are specific to a virtual assessment after a concussion. Specifically, the challenges with completing the virtual assessment due to screen intolerance and resulting symptom aggravation were a notable barrier to engaging in a virtual assessment. Depending on the time point in recovery, exposure to screens could be a very challenging barrier to overcome to complete a safe and effective assessment virtually. One strategy to address this issue, which was identified as a facilitator by participants, was implementing symptom management strategies (screen breaks and blue light glasses) so that the virtual assessment could be more tolerable. In addition, due to subtle deficits that are commonly experienced by people living with workplace concussions, virtual care approaches may not be currently adequate to capture these subtleties, and therefore, in-person care may be needed to identify issues that go beyond gross deficits. Furthermore, there are potential contradictions with the focus group data. For example, balance and the VOMS tool were selected as the vestibular measures that would work best in a virtual practice by clinicians. However, several issues were identified with these measures such as the challenges with visualizing subtle deficits associated with eye movements and balance when assessing virtually. Even with these challenges, the clinicians still believed that out of the measures identified in the surveys and discussed in the focus groups, these measures would be most appropriate for virtual assessments.

This work expands on some of the previous work on the clinician perspectives of virtual concussion care to outline barriers and facilitators to completing components of the physical examination [17]. Finally, this work reports on clinician-identified measures that will work best in a virtual context and identifies the barriers and facilitators associated with using these measures virtually so that the most appropriate measure for virtual administration can be selected. This work supports the identification of clinical measures to include in a virtual version of a concussion assessment, which will be further explored in a future planned evaluation study with the aim of documenting the psychometric properties of the measures. These measures will be included in a virtual assessment toolkit, which will be tested in a future study.

### Limitations

There are some important limitations to acknowledge in this work. The context of this work is within workplace injury, and all the patient participants who participated in the focus groups were experiencing persistent symptoms after injury at the time of their virtual assessment. Their experiences may be unique to those experiencing injuries occurring in the workplace, and therefore, the results may not be generalizable to people with concussions from other causes. Furthermore, only 27% of the patient participants were male, which indicates that the results of this study may be more reflective of female experiences. However, Merritt et al [50] reported that the prevalence of concussions is higher in female individuals, so the distribution of male individuals and female individuals in this study may be a close reflection of concussion occurrence in the working population. In addition, while attempts were made to include clinicians from various clinical fields (while focusing on professions that typically complete the physical examination), certain professions were not represented in the focus groups, such as neurology and occupational therapy. Finally, the selection of measures as most appropriate to use in a virtual assessment may be biased based on clinician experiences and practices. Clinician level of comfort with each clinical measure may have influenced their decisions.

### Conclusions

It is important to note that patients and clinicians identified limitations and challenges associated with virtual care. A hybrid approach to concussion assessment seems to be the most accepted model to assessment from the perspectives of both clinicians and people living with workplace concussions. This study demonstrates the acceptance and perceived value of virtual approaches in concussion assessment and therefore provides a rationale for further exploration of the psychometric properties of the virtual concussion assessment. There is an identified need to establish reliability and validity properties of the virtual concussion examination.

## Appendix

Multimedia Appendix 1 Standards for Reporting Qualitative Research checklist.

Multimedia Appendix 2 Barrier rankings.

Multimedia Appendix 3 Facilitator rankings.

Multimedia Appendix 4 Patient participant overarching themes, subthemes, and codes.

Multimedia Appendix 5 Clinician participant overarching themes, subthemes, and codes.

Multimedia Appendix 6 Patient subtheme definitions.

Multimedia Appendix 7 Clinician subtheme definitions.

## Abbreviations

VOMS: Vestibular/Ocular Motor Screening

## Data Availability

All data generated or analyzed during this study are included in this published manuscript and its multimedia appendices.
