# Supplementary material for: Barriers and Facilitators Associated With Remote Concussion Physical Assessments From the Perspectives of Clinicians and People Living With Workplace Concussions: Focus Group Study
Source: J Med Internet Res. 2024 Nov 13;26:e56158. doi: 10.2196/56158 (PMC11602758; doi:10.2196/56158)
Supplement: Multimedia Appendix 2 [file jmir_v26i1e56158_app2.docx]

**Appendix 2**

**Barrier Rankings**

The darker shades reflect barriers reported more frequently and lighter shades reflect barriers identified less frequently. White indicates that the barrier was not reported patient- or clinician-participants.

Ranking of barriers associated with virtual concussion assessment reported by patient- and clinician-participants

| **Barrier** | **Patient-Participant Rank** | **Clinician-Participant Rank** |
| --- | --- | --- |
| Accuracy/ completeness of physical exam |  |  |
| Communication and engagement |  |  |
| Technology and internet |  |  |
| Environmental set-up- Organizational Level |  |  |
| Triggering physical and emotional symptoms |  |  |
| Safety |  |  |
| Clinical process-related factors |  |  |
| Comfort |  |  |
| Environmental set-up- Individual Level |  |  |
| Visibility |  |  |
