# Supplementary material for: Barriers and Facilitators Associated With Remote Concussion Physical Assessments From the Perspectives of Clinicians and People Living With Workplace Concussions: Focus Group Study
Source: J Med Internet Res. 2024 Nov 13;26:e56158. doi: 10.2196/56158 (PMC11602758; doi:10.2196/56158)
Supplement: Multimedia Appendix 3 [file jmir_v26i1e56158_app3.docx]

**Appendix 3**

**Facilitator Rankings**

Ranking of facilitators associated with virtual concussion assessment reported by patient- and clinician-participants

| **Facilitator** | **Patient-Participant Rank** | **Clinician-Participant Rank** |
| --- | --- | --- |
| Support- Individual Level |  |  |
| Support- Organizational Level |  |  |
| Access |  |  |
| Format of delivered care and material |  |  |
| Symptom management |  |  |
| Use of resources |  |  |
| Environmental set-up |  |  |
| Use of measure to identify gross deficits |  |  |
