# Supplementary material for: Barriers and Facilitators Associated With Remote Concussion Physical Assessments From the Perspectives of Clinicians and People Living With Workplace Concussions: Focus Group Study
Source: J Med Internet Res. 2024 Nov 13;26:e56158. doi: 10.2196/56158 (PMC11602758; doi:10.2196/56158)
Supplement: Multimedia Appendix 4 [file jmir_v26i1e56158_app4.docx]

**Appendix 4**

**Patient Overarching Themes, Sub-Themes and Codes**
