# Supplementary material for: Barriers and Facilitators Associated With Remote Concussion Physical Assessments From the Perspectives of Clinicians and People Living With Workplace Concussions: Focus Group Study
Source: J Med Internet Res. 2024 Nov 13;26:e56158. doi: 10.2196/56158 (PMC11602758; doi:10.2196/56158)
Supplement: Multimedia Appendix 6 [file jmir_v26i1e56158_app6.docx]

**Appendix 6**

**Patient Sub-Theme Definitions**

| **Sub-theme** | **Definition** |
| --- | --- |
| Location and set-up at home | Having adequate space and an optimal set-up in the home environment |
| Use of functions available on the virtual platform | Use of recording function for later access to information |
| Introduction to technology and resources | Send information regarding what to expect with the virtual assessment and information about the assessment in advance |
| Integration and patient-selection of virtual | Having an in-person touchpoint prior to completing a virtual assessment and allowing the patient to use the device that will work best for them |
| Opportunity to involve more care team members | Opportunity to include more members involved in care at same appointment so that everyone s on the same page |
| Easy contact with clinician/technical support | Having someone that is available and easy to contact if needed for technical issues |
| Improved access | Improved access and easy access to care |
| Cost saving | Reduced cost associated with virtual assessment |
| Travel | No need to travel when attending virtual appointment |
| Screens trigger physical symptoms | Exposure to screens triggers concussive symptoms |
| Emotional | Feelings of isolation/depression when only attending virtual appointments |
| Challenges with communication | Difficulties communicating with clinicians through a screen |
| Challenges building rapport and connecting with clinician | Difficulties building rapport and connecting with clinicians through a screen |
| Able to push out of comfort zone with in-person | Feeling as though can push harder in terms of treatment and performance on tests in-person |
| Feelings of tests being safer in-person | Safety concerns (fall risk) while completing certain measures |
| Offering symptom management strategies | Being offered breaks and other symptom management strategies such as blue light glasses to aid with symptoms |
| Home support | Having a person at home or chair nearby for support |
| Healthcare system barriers | Longer wait times between clinicians with virtual care and different care teams use different virtual platforms |
| Issues/lack comfort with technology and internet | Challenges with connection due to internet issues |
| Format of required documents | Completing mandatory questionnaires online is more challenging than paper copies in-person |
| Incomplete evaluation/lack accuracy | Ability to complete a full evaluation and accurately assess and identify deficits is lacking |
| Lacking physical contact | Unable to complete hands on evaluation such as touching the neck |
| Sense of it being easier and more convenient at home | Convenience and ease associated with the virtual assessment, along with improved comfort at home |
