# Supplementary material for: Barriers and Facilitators Associated With Remote Concussion Physical Assessments From the Perspectives of Clinicians and People Living With Workplace Concussions: Focus Group Study
Source: J Med Internet Res. 2024 Nov 13;26:e56158. doi: 10.2196/56158 (PMC11602758; doi:10.2196/56158)
Supplement: Multimedia Appendix 7 [file jmir_v26i1e56158_app7.docx]

**Appendix 7**

**Clinician Sub-Theme Definitions**

| **Sub-Theme** | **Definition** |
| --- | --- |
| Lack administrative support | Administrators are not always present to aid with technical difficulties |
| Time | Limited time to complete an assessment |
| Symptom triggering | Exposure to screens triggers concussive symptoms |
| Patient comfort and preference | Lack of patient comfort with a virtual assessment and preference for in-person for certain aspects of care |
| Psychological impact | Clinician-stress associated with dealing with technical issues in a virtual assessment |
| Challenges with communication and connection | Difficulties communicating with patients and building rapport through a screen |
| Lack accuracy | Ability to accurately assess and identify deficits is lacking |
| Environmental and patient set-up | Home environment of the patient (space, ability to position oneself on camera) |
| Visibility | Challenges seeing a patient on camera |
| Safety | Safety concerns (fall risk) while completing certain measures |
| Unable to assess certain conditions and subtleties | Difficulties assessing certain conditions such as vertigo and difficulties assessing subtleties such as nystagmus |
| Unable to complete assessment in entirety | Unable to complete hands on evaluation such as palpating the neck or completing a fundoscopy exam |
| Camera and device limitations | Limitations associated with the device the patient is using (phone) and camera quality issues |
| Technical and Internet issues | Challenges with connection due to internet issues |
| Support | Having a person at home or chair nearby for support |
| Use of resources to complete assessment | Use of mobile applications or videos to support the virtual assessment |
| Introduction to technology and resources | Send information regarding what to expect with the virtual assessment and information about the assessment in advance |
| Environmental and patient set-up | Having adequate space and an optimal set-up in the home environment |
| Use of measure as global screen and rely on subjective information | Using clinical measures as a screen of global deficits rather than as a measure of specific deficits and relying on subjective information provided by the patient rather than objective information |
| Integration and patient-selection of virtual | Having an in-person touchpoint prior to completing a virtual assessment and allowing the patient to use the device that will work best for them |
| Access | Improved access and easy access to care |
| Sense of it being easier, more convenient and more comfortable | Convenience and ease associated with the virtual assessment, along with improved patient comfort at home |
